# Down-regulation of *miR-7-5p* and *miR-548ar-5p* predicts malignancy in indeterminate thyroid nodules negative for *BRAF* and *RAS* mutations

**DOI:** 10.1007/s12020-022-03034-7

**Published:** 2022-03-26

**Authors:** Elisabetta Macerola, Anello Marcello Poma, Agnese Proietti, Teresa Rago, Rossana Romani, Paola Vignali, Clara Ugolini, Liborio Torregrossa, Alessio Basolo, Ferruccio Santini, Fulvio Basolo

**Affiliations:** 1 University of Pisa, Department of Surgical, Medical, Molecular Pathology and Critical Area – via Savi, 10 56126 Pisa, Italy; 2 University of Pisa, Department of Clinical and Experimental Medicine – via Roma, 67 56126 Pisa, Italy; 3 University Hospital of Pisa – via Roma, 67 56126 Pisa, Italy

**Keywords:** Thyroid nodules, Papillary thyroid carcinoma, miRNA, mir-7-5p, mir-548ar-5p

## Abstract

**Purpose:**

The value of molecular markers in refining preoperative risk assessment of indeterminate thyroid nodules is being widely investigated. MicroRNAs (miRNA) are emerging as promising biomarkers for diagnostic and prognostic purposes. The aim of this study is to identify miRNAs specifically deregulated in mutation-negative indeterminate thyroid nodules.

**Methods:**

Ninety-eight nodules preoperatively diagnosed as TIR 3A or TIR 3B with available histological diagnosis of follicular adenoma (FA), noninvasive follicular neoplasm with papillary-like nuclear features (NIFTP), and follicular variant papillary thyroid carcinoma (FV-PTC) have been retrospectively selected. Mutations in *BRAF* and *RAS* genes have been tested in all samples by real-time PCR; miRNAs were purified from cytology slides of 60 samples; expression analysis of 798 miRNAs was measured by the nCounter system.

**Results:**

Point mutations in *BRAF* and *RAS* genes were detected in 32 out of 98 nodules (32.7%), the majority of which in FV-PTCs. Differential expression of miRNA in wild-type nodules highlighted that two miRNAs, namely *miR-7-5p* and *miR-548ar-5p*, were downregulated in FV-PTCs compared to FAs. The combined expression of these miRNAs, tested by ROC analysis, showed an area under the curve of 0.79. Sensitivity and negative predictive value were high both in wild-type (93% and 92%, respectively) and in mutated nodules (94% and 85%, respectively).

**Conclusion:**

The analysis of *miR-7-5p* and *miR-548ar-5p* expression in indeterminate thyroid nodules demonstrated a promising value in ruling out malignancy.

## Introduction

The clinical management of patients with cytologically indeterminate thyroid nodules represents a challenging issue. Ultrasound (US) examination is an essential instrument for the initial evaluation of nodules and also for patient follow-up. In order to better characterize thyroid nodules, several US-based stratification systems have been, but their overall performance in the setting of indeterminate cytology is poor [1]. Indeterminate nodules, namely TIR3A and TIR3B according to the Italian System [2] and classes III and IV according to the Bethesda system [3], generally prove to be follicular architecture lesions after surgery, most frequently follicular adenoma, noninvasive follicular neoplasm with papillary-like nuclear features (NIFTP), follicular variant papillary thyroid carcinoma (FV-PTC), and rarely follicular thyroid carcinoma (FTC) [2, 4]. Malignant lesions arising as indeterminate nodules are generally low-risk tumors for which, in most cases, lobectomy represents a definitive treatment [5–7]; patients rarely need re-operation for completion thyroidectomy. However, on histological diagnosis the rate of benign lesions is high, and most patients are subjected to unnecessary surgical procedures. Therefore, indeterminate nodules should be characterized as well as possible preoperatively, so as to be able to select the most appropriate treatment strategy.

The use of ancillary molecular tests including—at least the analysis of the most frequently mutated genes—can improve the diagnostic accuracy of cytology. In detail, the presence of gene mutations in *BRAF* and *RAS* genes increases the risk of malignancy up to 80% [8, 9]. Recently, scientific societies have indicated that a molecular testing with the so-called 7-gene panel can effectively integrate the evaluation of indeterminate cytology [3, 10]. According to the current European Thyroid Association (ETA) and American Thyroid Association (ATA) guidelines, molecular testing performed on Bethesda III and IV nodules can supplement their clinical assessment by providing information useful to refine the risk of nodule malignancy [11, 12].

If the presence of a genetic mutation is rather specific for malignancy, a negative result must be considered as not informative, owing to the low sensitivity of *BRAF* and *RAS* molecular testing. MicroRNAs (miRNAs) are emerging as biomarkers whose deregulated expression is associated with pathological conditions. Several miRNAs have been investigated as possible diagnostic markers in indeterminate thyroid cytology, and some PTC-specific markers have been identified so far [13, 14]. Some of these miRNAs not only show upregulation in PTC compared to normal tissue or benign thyroid lesions, but they have also been correlated to patient prognosis. For instance, overexpression of *miR-146*, *miR-221*, and *miR-222* have been associated with a higher risk of recurrence in PTC patients [15]. However, miRNAs specifically deregulated in benign and malignant follicular-patterned lesions are still under investigation.

The aim of this study is to identify miRNAs useful to the differential diagnosis of indeterminate thyroid nodules; specifically, we analyzed nodules with homogeneous cytological diagnosis (TIR3A and TIR3B), absence of point mutations in *BRAF* and *RAS* genes and available histological diagnosis of benign and malignant low-risk thyroid lesions, namely follicular adenoma, NIFTP and follicular variant of PTC.

## Materials and methods

### Study cohort

In the period January 2018 – December 2020 institutional archives were searched for cytology cases matching the following criteria: diagnosis of TIR3A or TIR3B; availability of cytological slides for pathological revision; availability of histological diagnosis of follicular-patterned thyroid lesions, specifically follicular adenoma, follicular variant PTC, or NIFTP. Cytological slides were retrieved and carefully revised by three cyto-pathologists with experience in thyroid pathology. One cytological smear stained with Papanicolaou per case was selected for miRNA isolation; at least ten groups of follicular cells had to be present on a single slide; more than six slides per case had to be available, so that sufficient material could be left for future evaluation. Coverslips were removed after 24 h immersion in xylene, and the slides were then rehydrated through a descending series of ethanol to water. miRNA extraction was performed after manually dissecting areas containing follicular cells, according to the manufacturer’s instruction (miRNeasy FFPE kit, Qiagen, Hilden, Germany). Total RNA quantity and quality were assessed by the spectrophotometer XPose (Trinean, Gentbrugge, Belgium). A standardized protocol for the simultaneous recovery of miRNA and DNA was not available, therefore, to avoid the use of an additional cytological slide, mutation analysis was conducted on DNA purified from the paraffin tissue specimens corresponding to the analyzed nodules. To allow a selective dissection of material, areas containing tumor cells were marked on the hematoxylin-eosin slides; the percentage of tumor cells was also determined. After standard deparaffination of three 10 µm-thick unstained tissue slides per case, DNA was extracted by using the Qiamp DNA Mini Kit (Qiagen).

### Ultrasound evaluation

Neck ultrasound was performed in all patients by Real-time instrument (Technos, Esaote Biomedica, Genova, Italy) by using a 7.5 MHz linear transducer [16] in the context of a dedicated center for thyroid nodule pathology (Center of Endocrinology, University Hospital of Pisa). Nodules were scored with the EU-TIRADS system as recommended by the ETA [17].

### Analysis of *BRAF* and *RAS* mutations

Samples were first tested by high resolution melt analysis (HRMA) by using the Type It HRM master mix (Qiagen) to detect mutations in *BRAF* exon 15, *NRAS* codon 61, and *HRAS* codon 61. Samples with altered melt profile were sequenced on an ABI 3130 genetic analyzer. A commercial real-time PCR kit was used on wild-type samples to assess the presence of mutations in codons 12 and 13 of *NRAS* and in codons 12, 13, and 61 of *KRAS* (EasyPGX Ready THYROID kit, Diatech Pharmacogenetics, Jesi, AN, Italy). *TERT* promoter mutations, indicative of high-risk histology and thus virtually absent in this series, were not tested.

### Analysis of miRNA expression by nCounter

The analysis of miRNA was performed by using the nCounter Human v3 miRNA Expression Assay (nanoString Technologies, Seattle, WA, USA), targeting 828 miRNAs, on the nCounter system (nanoString Technologies). The panel includes probe pairs for 798 endogenous miRNAs and for 30 controls, comprising positive, negative and ligation controls. Samples were prepared for hybridization following the manufacturer’s instructions. After purification and digital counting, raw data were analyzed by the nSolver analysis software v4.0 (nanoString Technologies). Biological normalization was performed by using the mean expression of the top 100 expressed miRNAs. After adding 0.1 to log2 normalized counts to avoid dealing with log of 0, in order to select and analyze only the miRNAs highly expressed in all samples and those expressed with a large variation among samples, miRNAs were filtered according to the following criteria: (a) miRNAs with mean log2 expression above 4 were selected; (b) of the miRNAs with mean log2 expression below 4, only those with variance higher than or equal to 1.5-fold the mean were considered.

### Analysis of *miR-7-5p* and *miR-548ar-5p* expression by RT-PCR

Reverse transcription PCR (RT-PCR) was performed on a subset of 17 samples already analyzed by the nCounter. These samples were all negative for gene mutations, and were chosen on the basis of RNA availability: the very same RNA sample was used to avoid any source of variability. The *miR-7-5p* and *miR-548ar-5p* expression was tested by real-time RT-PCR along with *U6* small nuclear RNA (*RNU6-1*) as reference by using the TaqMan Assay system (Thermofisher Scientific, Waltham, MA, USA). RT was performed by adding target-specific primers with 10 ng of input RNA according to the manufacturer’s protocol. The experiments were conducted in triplicate on a RotorGene Q instrument (Qiagen).

### Statistical analysis

Principal component analysis (PCA) was conducted by using the PCAtools Bioconductor package v.2.4.0. Differential miRNA expression analysis was performed following the procedures of the limma Bioconductor package v.3.48.1. In detail, FAs were used as baseline and the Benjamini-Hochberg method was employed to adjust the *P*-values. The correlation between miRNAs was tested by the Pearson’s method. The miR-score was built by averaging the expression level of miRNAs with a *P*-value below 0.05 and an absolute log2 fold change (FC) higher than 2. Receiver operating characteristics (ROC) curves analysis was used to assess diagnostic performance, following the procedures of the pROC R package v.1.17.0.1. Confidence intervals were computed by 2000 bootstrap replicates. For the analysis, NIFTPs were considered malignant since they required surgical excision.

RT-PCR data were analyzed by the ddCt Bioconductor package v.1.50.0; amplification data in the form of threshold cycles (Ct) were exported and relative expression was computed using the 2-ΔΔCt method [18]. The comparison of the expression levels measured by the RT-PCR and the nCounter method was performed by the Passing-Bablok regression so as to avoid any kind of assumption on data distribution, and following the procedures of the mcr R package v.1.2.2. The nCounter was set as reference method, while RT-PCR was used as the test method.

All analyses were performed in R environment (https://www.r-project.org, v.4.1.0, last accessed November 12, 2021).

## Results

Ninety-eight cytological slides were included in the cohort study. Details on the characteristics of the nodules are shown in Table 1. Analyses of miRNA expression and US characteristics were conducted in subgroups of patients, as shown in Table 1. No differences in terms of cytological diagnosis, mutations nor histology were present between these subgroups and the entire sample cohort.

**Table 1:** Cytological, molecular and histological characteristics of all samples *n* = 98, samples subjected to miRNA expression analysis (*n* = 60) and nodules whose US evaluation was available (*n* = 58)

		All cases *n* = 98	miRNA analysis *n* = 60	US data *n* = 58
*Age*	mean ± SD	48 ± 15	50 ± 15	49.1 ± 15.3
*Gender*	F	59 (60.2%)	38 (63.3%)	34 (58.6%)
	M	39 (39.8%)	22 (36.7%)	24 (41.4%)
*Cytology*	TIR3A	63 (64.3%)	37 (61.7%)	40 (69.0%)
	TIR3B	35 (35.7%)	23 (38.3%)	18 (31.0%)
*Mutational status*	wild-type	66 (67.3%)	40 (66.7%)	40 (69.0%)
	mutated	32 (32.7%)	20 (33.3%)	18 (31.0%)
*Histology*	FA	41 (41.8%)	24 (40.0%)	24 (41.4%)
	FV-PTC	47 (48.0%)	30 (50.0%)	26 (44.8%)
	NIFTP	10 (10.2%)	6 (10.0%)	8 (13.8%)

Abbreviations: *US* Ultrasound, *FA* Follicular adenoma, *FV-PTC* Follicular variant papillary thyroid carcinoma, *NIFTP* Noninvasive follicular neoplasm with papillary-like nuclear features

### Ultrasound evaluation

Presurgical US evaluation was available for 58 out of 98 nodules (59.2%). According to the EU-TIRADS categories (Table 2), the majority of nodules were benign (EU-TIRADS 2, 52 out of 58, 89.7%); six nodules were low-risk (EU-TIRADS 3, 6 out of 58, 10.3%); no nodules with intermediate or high-risk (EU-TIRADS 4 and 5) were present.

**Table 2:** EU-TIRADS classification of the 58 nodules whose US evaluation was available. Cases have been also divided according to the mutational status

US characteristics		All nodules *n* = 58	Wild-type nodules *n* = 40	Mutated nodules *n* = 18
EU-TIRADS category	2	52 (89.7%)	36 (90.0%)	16 (88.9%)
	3	6 (10.3%)	4 (10.0%)	2 (11.1%)

Abbreviations: *US* Ultrasound, *EU-TIRADS* European thyroid imaging and reporting data system

### Mutational status and histological diagnosis of nodules

Tumor cell fraction in dissected tissue areas was higher than 60% in all cases. Of the 98 nodules, 32 were positive for genetic mutations (32.6%), which were all *RAS*-like mutations. *NRAS* mutation was detected in 18 samples, *HRAS* mutation in seven and *KRAS* mutation in four. Non-V600E *BRAF* mutations were detected in three nodules (one p.K601E, one p.K601N, one p.T599del). Mutations were present in 19 out of 63 TIR3A (30.2%) and in 13 out of 35 TIR3B nodules (37.1%). The correlation between cytology and histology demonstrated that out of 63 TIR 3A nodules, 34 were FA (54%), 23 were FV-PTC (36.5%), and six were NIFTP (9.5%). Out of the 35 TIR 3B nodules, seven were FA (20%), 24 were FV-PTC (68.6%), and four were NIFTP (11.4%). On histological examination, only two FV-PTCs out of 47 (4.2%) presented with minimal extrathyroidal extension. According to histological diagnosis, mutations were found in two out of 41 FAs (4.9%), 26 out of 47 FV-PTCs (55.3%) and four out of ten NIFTPs (40%) (Table 3).

**Table 3:** Details on correlation between cytology, histology and molecular alterations in all the 98 nodules

	*Histology*					
*Cytology*	FA *n* = 41		FV-PTC *n* = 47		NIFTP *n* = 10	
TIR 3A *n* = 63	34	*Mutated*	23	*Mutated*	6	*Mutated*
		2		14		3
TIR 3B *n* = 35	7	*Mutated*	24	*Mutated*	4	*Mutated*
		0		12		1

Abbreviations: *FA* Follicular adenoma, *FV-PTC* Follicular variant papillary thyroid carcinoma, *NIFTP* Noninvasive follicular neoplasm with papillary-like nuclear features

### miRNA expression analysis

The nCounter analysis was conducted on the 60 samples that met criteria for miRNA extraction, including 40 mutation-negative and 20 mutation-positive cases, distributed as shown in Table 1. In detail, miRNA extraction was not performed in nine cases with an insufficient number of follicular cells, and in 29 cases for which there was a low number of slides available.

Of the 60 analyzed samples, two failed the software quality check on account of poor RNA content in one case and failure of internal positive control in the other. Data analysis was then conducted on 58 samples. After data normalization and filtering, 99 miRNAs were selected for downstream analyses.

**Fig. 1:**
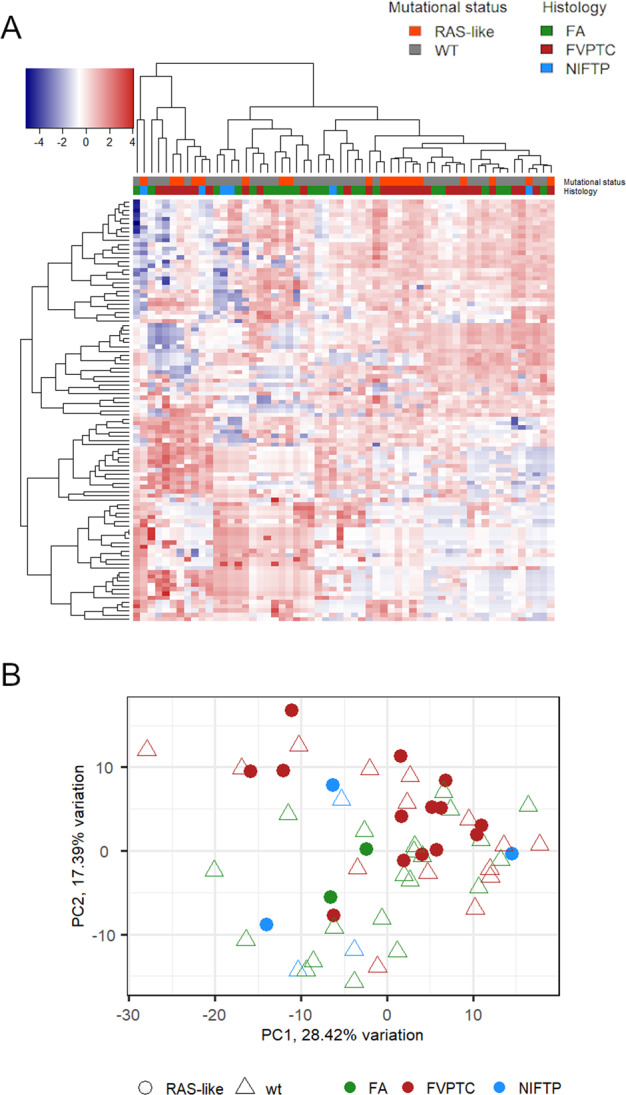
Heatmap showing the expression levels of the 99 expressed miRNAs in all the analyzed samples (*n* = 58) (**A**); in this unsupervised cluster, each row represents a miRNA, each column represents a sample. Color intensity is related to expression level (high miRNA expression, red; low miRNA expression, blue). Colored sidebars on the top of cluster label samples based on their histology and mutational status. Principal component analysis of the 99 expressed miRNAs (**B**). Samples have been labeled according to mutational status and histology. RAS-like mutated cases are indicated with circles, wild-type samples with triangles; different colors represent histological diagnosis of FA (green), FV-PTC (red), and NIFTP (light blue)

The heatmap in Fig. 1A shows the expression levels of the 99 miRNAs across all the analyzed samples. As highlighted by the colored sidebars, there was no clear clusterization of samples. The PCA in Fig. 1B illustrates the distribution of samples according to miRNA expression in relation to histology and mutational status. PC1 and PC2 cumulatively accounted for almost 50% of total variation. Although a certain clustering of FV-PTC is visible, there is partial overlapping of the samples according to histology and mutational status.

**Fig. 2:**
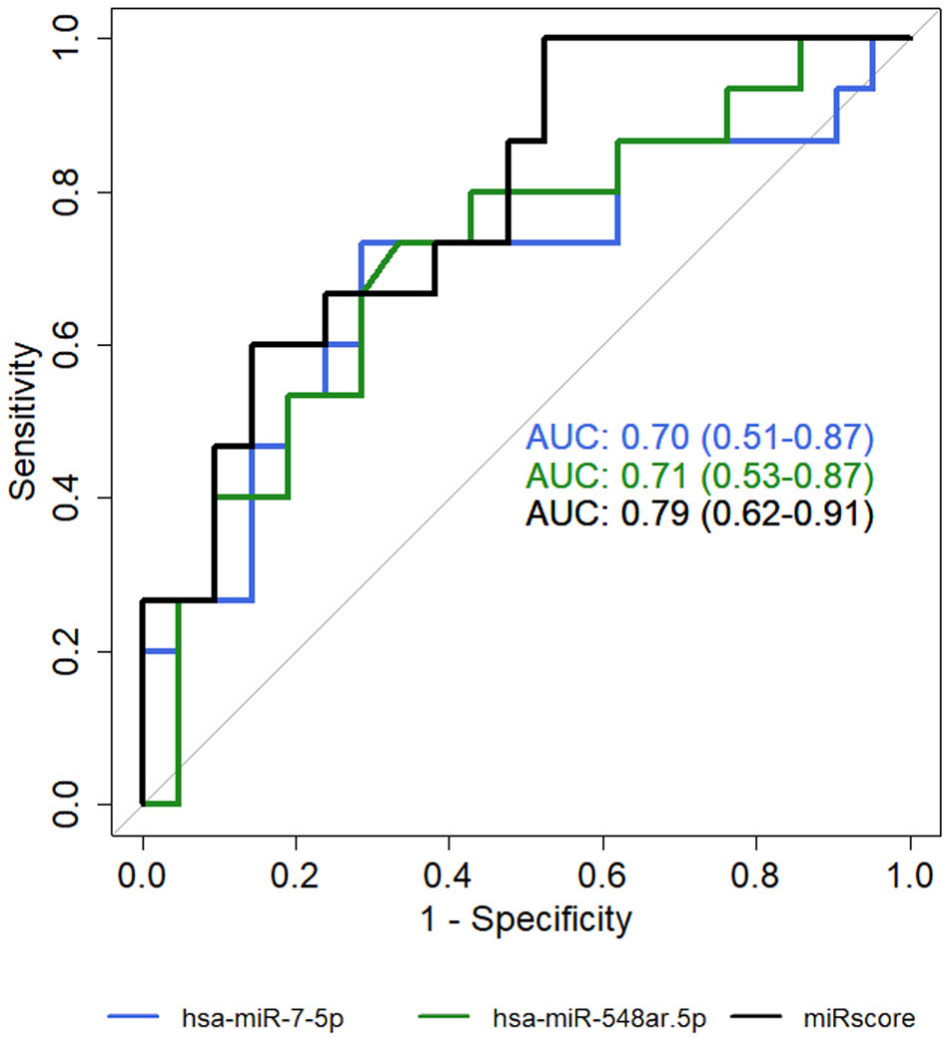
Area under the curve (AUC) of *miR-7-5p*, *miR-548ar-5p,* and miR-score in wild-type samples

**Fig. 3:**
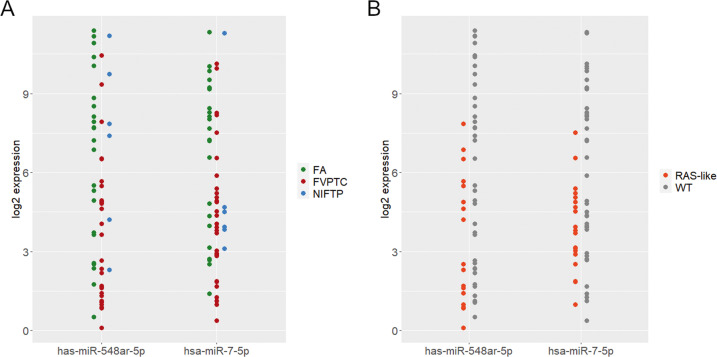
Expression of *miR-7-5p* and *miR-548ar-5p* in all samples (mutated and wild-type). In (**A**) samples have been labeled according to their histological category. There is a trend for FAs to upregulate *miR-7-5p* and *miR-548ar-5p*. In (**B**) samples can be distinguished based on their mutational status. Mutation-positive samples seem to repress the expression of the two miRNAs

Analysis of the differential miRNA expression was conducted comparing the 21 wild-type FAs versus the 15 wild-type FV-PTCs. The results showed that none of the considered miRNAs had a significantly different expression between the two groups after *P*-value correction for multiple comparisons. Two miRNAs, *miR-7-5p* and *miR-548ar-5p*, were downregulated in FV-PTCs compared to FAs (*P*-value 0.01 and 0.02, respectively) with an absolute value of log FC higher than 2. These miRNAs were then selected for ROC analysis. The combination of both miRNAs was also tested (miR-score), since their expression was not correlated (R = 0.08; *P*-value 0.65). The results of ROC analysis for *miR-7-5p* and *miR-548ar-5p* and their combination in the 21 wild-type FAs versus the 15 wild-type FV-PTCs appear in Table 4 and Fig. 2. The area under the curve (AUC) was higher when the miR-score was used. The AUC and overall performance of the miR-score were also calculated in all the tested samples along with those of point mutations (Table 5). The *miR-7-5p* and *miR-548ar-5p* expression levels in all the samples can be observed in the dot plots in Fig. 3. Figure 3A shows the expression levels in all samples according to the histological category: FV-PTCs tend to down-regulate these miRNAs compared to FAs, while NIFTPs are distributed variously. As illustrated in Fig. 3B, samples that are positive for mutations clearly show a downregulation of *miR-7-5p* and *miR-548ar-5p*.

**Table 4:** AUC, sensitivity, specificity, PPV, NPV, and accuracy of selected miRNAs obtained by ROC analysis in wild-type FA and FV-PTC samples

	AUC (95% CI)	Sensitivity (95% CI)	Specificity (95% CI)	PPV (95% CI)	NPV (95% CI)	Accuracy (95% CI)
*miR-7-5p*	0.69 (0.49–0.86)	0.73 (0.40–0.93)	0.76 (0.48–1)	0.69 (0.53–1)	0.79 (0.67–0.95)	0.75 (0.61–0.86)
*miR-548ar-5p*	0.71 (0.53–0.89)	0.73 (0.40–1)	0.76 (0.38–1)	0.67 (0.50–1)	0.80 (0.67–1)	0.72 (0.58–0.86)
miR-score	0.79 (0.63–0.92)	0.93 (0.53–1)	0.71 (0.38–1)	0.68 (0.54–1)	0.92 (0.72–1)	0.75 (0.64–0.89)

Abbreviations: *AUC* Area under the curve, *CI* Confidence interval, *PPV* Positive predictive value, *NPV* Negative predictive value

**Table 5:** Test performance of *BRAF* and *RAS* mutations and miR-score

	*n* samples	AUC (95% CI)	Sensitivity (95% CI)	Specificity (95% CI)	PPV (95% CI)	NPV (95% CI)	Accuracy (95% CI)
Mutations	98	0.74 (0.66–0.81)	0.53 (0.40–0.67)	0.95 (0.88–1)	0.94 (0.85–1)	0.59 (0.53–0.67)	0.70 (0.62–0.79)
miR-score	58	0.77 (0.64–0.88)	0.94 (0.86–1)	0.44 (0.22–0.61)	0.72 (0.65–0.79)	0.85 (0.61–1)	0.74 (0.64–0.83)

Abbreviations: *AUC* Area under the curve, *CI* Confidence interval, *PPV* Positive predictive value, *NPV* Negative predictive value

**Fig. 4:**
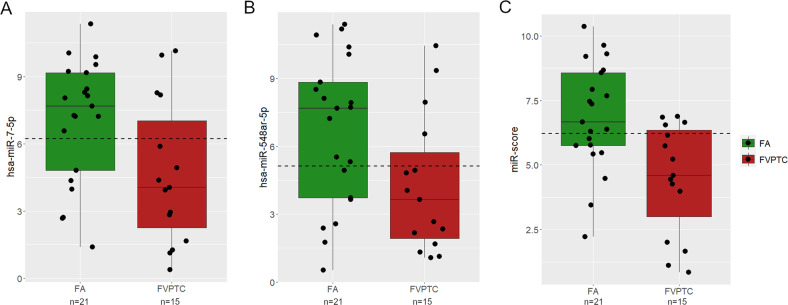
Expression of *miR-7-5p* (**A**), *miR-548ar-5p* (**B**), and their combination (miR-score, **C**) in mutation-negative FAs and FV-PTCs. Dashed lines represent the expression cutoffs identified by ROC analysis

The boxplots in Fig. 4 show the expression of *miR-7-5p*, *miR-548ar-5p,* and their combination in wild-type samples (*n* = 36), according to the expression cutoffs identified by the Youden J statistics. NIFTPs were not included in Fig. 4 because there were only three wild-type cases. The three wild-type NIFTPs had miRNA expression above miR-score cutoff; on the contrary, the three mutated NIFTPs showed a miR-score below the set threshold.

### Analysis of *miR-7-5p* and *miR-548ar-5p* expression by RT-PCR

**Fig. 5:**
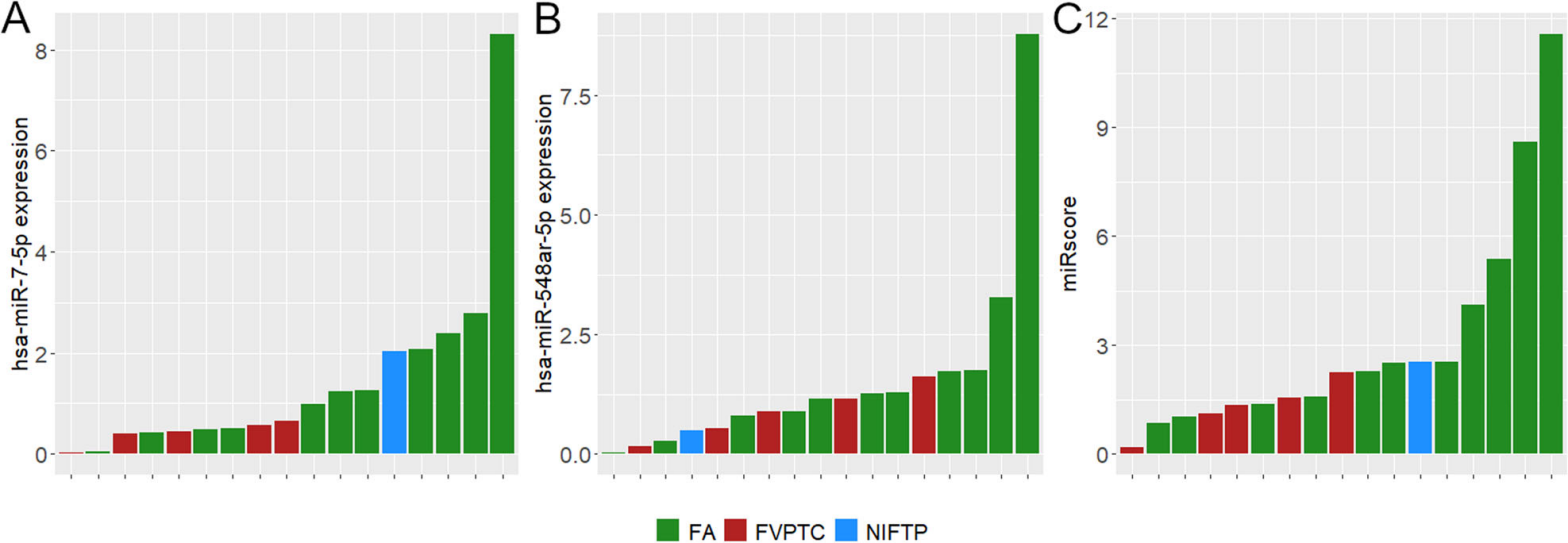
Histograms show the relative expression levels of *miR-7-5p* (**A**), *miR-548ar-5p* (**B**), and miR-score (**C**) measured by RT-PCR in 17 samples

The 17 analyzed samples were distributed as follows: 11 FAs, 5 FV-PTCs and 1 NIFTP. All samples showed amplification curves for the analyzed targets. Expression levels of *miR-7-5p*, *miR-548ar-5p* and miR-score can be observed in Fig. 5. The AUC and the test performance values obtained for these 17 samples and only based on RT-PCR expression data are reported in Table 6.

**Table 6:** ROC analysis results in the subset of 17 wild-type samples analyzed for *miR-7-5p* and *miR-548ar-5p* by RT-PCR. The reported values are referred exclusively to miRNA expression levels obtained by RT-PCR

	AUC (95% CI)	Sensitivity (95% CI)	Specificity (95% CI)	PPV (95% CI)	NPV (95% CI)	Accuracy (95% CI)
*miR-7-5p*	0.73 (0.47–0.94)	0.83 (0.33–1)	0.73 (0.27–1)	0.60 (0.43–1)	0.91 (0.73–1)	0.76 (0.53–0.94)
*miR-548ar-5p*	0.70 (0.42–0.94)	0.83 (0.50–1)	0.73 (0.27–1)	0.60 (0.42–1)	0.91 (0.75–1)	0.76 (0.53–0.94)
miR-score	0.74 (0.48–0.95)	1 (0.50–1)	0.73 (0.27–1)	0.60 (0.43–1)	0.92 (0.72–1)	1 (0.77–1)

Abbreviations: *AUC* Area under the curve, *CI* Confidence interval, *PPV* Positive predictive value, *NPV* Negative predictive value

**Fig. 6:**
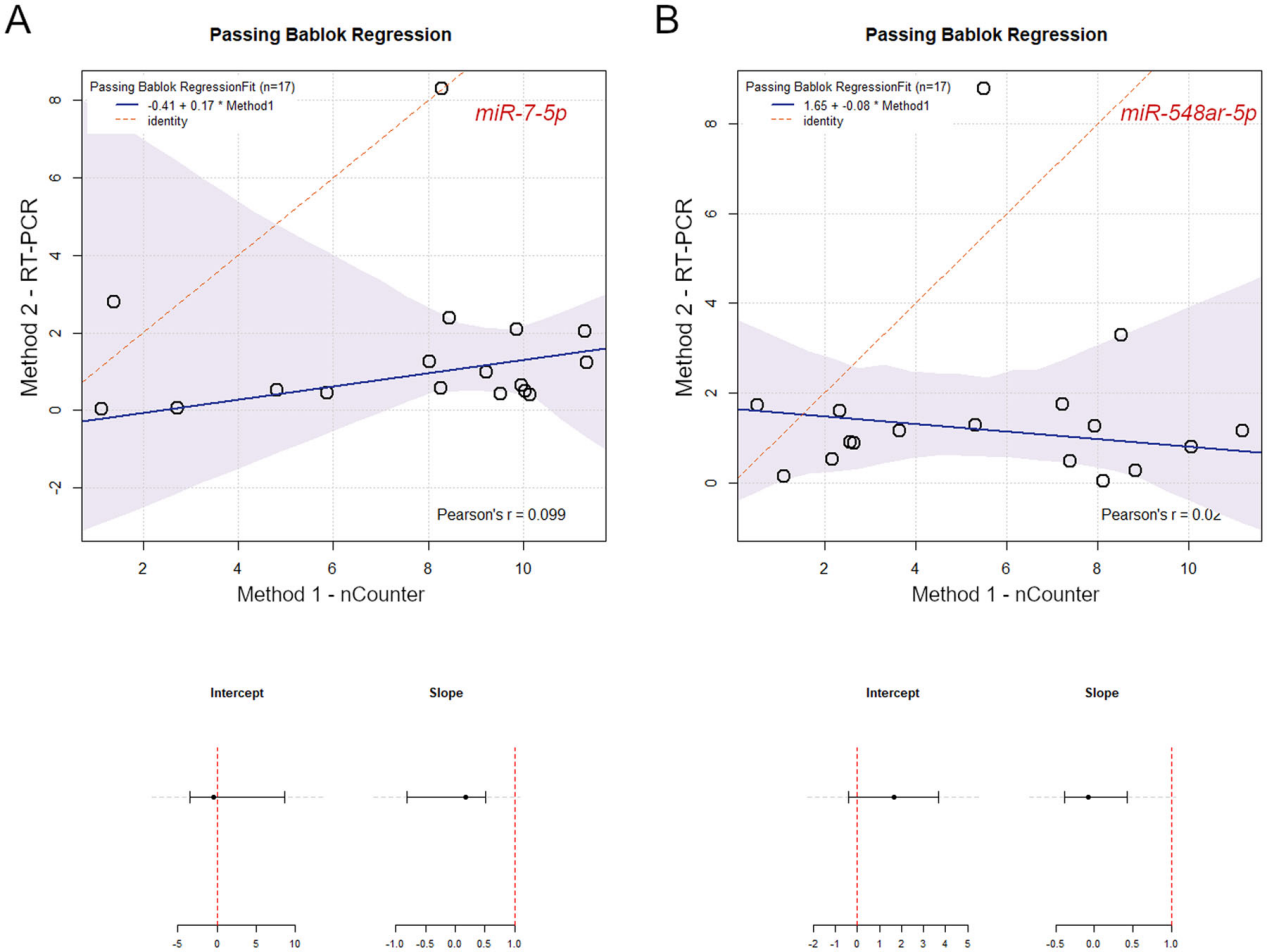
Passing-Bablok regression for method comparison. The graphs show the correlation between expression levels of *miR-7-5p* (**A**) and *miR-548ar-5p* (**B**) measured by nCounter (*x*-axis) and RT-PCR analysis (*y*-axis). On the bottom, slope and intercept values and related confidence interval (bars) are shown. For both miRNAs, intercept confidence interval does not include 1, indicating the presence of a proportional error in the measurement

According to the Passing-Bablok regression, the intercept for *miR-7-5p* was −0.41 (95% CI −3.45 – 8.67) and the slope was 0.17 (95% CI −0.81–0.51); the intercept for *miR-548ar-5p* was 1.65 (95% CI −0.41–3.66) and the slope was −0.08 (95% CI −0.39–0.42). With regard to both miRNAs, 1 was not included in the CI of the slope, indicating the existence of a proportional difference between the two measurement methods (Fig. 6).

## Discussion

The role of molecular testing in improving the presurgical evaluation of indeterminate thyroid nodules has been widely recognized. As a matter of fact, a better characterization of indeterminate cytology can be useful to avoid both diagnostic surgery and two-step surgery.

A recent meta-analysis has investigated the diagnostic performance of the main commercial tests dedicated to thyroid cytology, including ThyroSeq v3 and Afirma Genome Sequencing Classifier (GSC) [19]. These two tests were confirmed as having a good NPV, higher than 90%. The performance of a molecular test is strictly related to the risk of malignancy observed in each specific institution for indeterminate thyroid nodules. This is in turn related to the cytopathologist’s experience, to US evaluation, and also to the ways in which all this information is integrated and to how it influences the clinical judgment. For instance, a molecular test with a high NPV could be suitable for an institution showing a low prevalence of cancer among indeterminate nodules. In that case, the reduction of unnecessary diagnostic thyroidectomies would be considerable, and the costs related to molecular tests would be largely justifiable. Both ThyroSeq v3 and Afirma GSC tests are offered by private companies, but the costs are not covered by the National Health Systems. On the other hand, in many molecular pathology laboratories, an approach based on a reduced number of markers like the 7-gene panel, represents a practical and cost-effective option.

US scoring systems, such as the EU-TIRADS system, are helpful clinical tools for the selection of nodules deserving FNA and for the risk assessment of thyroid nodules. A higher US score is generally associated with a higher risk of malignancy. However, cytologically indeterminate nodules often do not show worrisome US characteristics, so that the performance of the EU-TIRADS system is overall poor [1, 20, 21].

The aim of this study was to find miRNAs able to differentiate benign from malignant follicular-architecture lesions in *BRAF*- and *RAS*-negative nodules. Analysis of miRNA expression analysis was conducted directly on the cytological slides, which represent the only diagnostic material available in the presurgical setting.

By comparing wild-type FAs versus wild-type FV-PTCs, none of the 99 expressed miRNAs showed significant differences after statistical correction for multiple comparisons caused by low statistical power. However, *miR-7-5p* and *miR-548ar-5p* were downregulated in FV-PTC with *P*-value <0.05 and fold change >2. ROC analysis for these two miRNAs demonstrated that their combination (miR-score) best performed in terms of AUC (0.79), sensitivity (93%) and NPV (92%) in wild-type cases. To confirm the potential value of these markers, the miR-score was also tested in all the samples analyzed with the nCounter (including mutated cases) and was then compared with the performance of gene mutations. As shown in Fig. 3 and Table 5, the miR-score displayed a better performance in terms of AUC, reaching a sensitivity of 94% and a NPV of 85%. It should also be noted that, despite a considerably high sensitivity, NPV was affected by the high prevalence of malignancy in our series (48%), as a result of the selection criteria adopted.

*miR-7-5p* has been previously described as suppressor miRNA across multiple cancer types [22], including lung, colon, and breast cancer. In particular, *miR-7-5p* acts as tumor suppressor in signaling pathways with a crucial role in cancer (EGFR/MAPK, PI3K/Akt, Wnt, and others), inhibiting cell survival, proliferation, and migration.

In thyroid cancer, *miR-7-5p* has been described as downregulated in PTC compared to benign thyroid tissue [23, 24]. In particular, an intriguing result obtained by Jahanbani et al. raised the possibility that *miR-7-5p* could serve as a NIFTP-specific marker [25]. The authors found that NIFTPs showed significant downregulation of this miRNA compared to thyroid hyperplasia; however, downregulation of miR-7-5p was also observed also in PTCs. In our series the *miR-7-5p* and *miR-548ar-5p* (miR-score) expression in NIFTPs was variable, likely depending on their mutational status: the miR-score was higher in three wild-type NIFTPs, while it was below the cutoff in three *RAS*-mutated NIFTPs. It can be hypothesized that wild-type NIFTPs maintain *miR-7-5p* and *miR-548ar-5p* expression similarly to FAs, while mutated NIFTPs tend to lose these miRNAs similarly to FV-PTCs. However, the number of NIFTP cases in this series makes it difficult to draw definitive conclusions in this regard.

It is important to note that the results herein provided represent the first evidence—obtained directly from fine-needle aspiration cytology specimens – that *miR-7-5p* is downregulated in follicular-patterned thyroid malignancies.

Only a few reports have investigated the role of *miR-548ar-5p* in cancer. This miRNA has hundreds of predicted target genes. It belongs to the *miR-548* family, which is involved in several biological processes, including MAPK and Wnt signaling pathways [26]. For instance, *miR-548-3p* has been described as significantly downregulated in breast cancer; its tumors suppressor role has also been confirmed in vitro [27]. Further studies are needed to investigate the role of *miR-548ar-5p* in thyroid tumors and to experimentally validate its target genes.

Nodules with high cyto-histological, US, and molecular similarity were selected for this cohort study. Indeed, all nodules presented with a cytologically indeterminate diagnosis and with benign or low-risk US features; histologically, all nodules were low-risk follicular-architecture thyroid neoplasms. In this context, the presurgical characterization of nodules should be as accurate as possible, in order to select the optimal clinical and surgical management of patients. This work demonstrates that the analysis of two miRNAs is highly capable of ruling-out malignancy both in *BRAF*/*RAS*-mutated and in wild-type nodules.

This study has some limitations. Molecular testing was limited to BRAF and RAS genes. Screening for additional RAS-like alterations, for instance, EIF1AX mutations and PPARG fusions, would have added important pieces of information and strengthened our findings. Moreover, miRNA expression analysis and mutational screening were conducted on nucleic acids purified from different types of material, i.e., cytological smear and paraffin tissue, respectively. This depended on the unavailability of an optimized protocol for the simultaneous recovery of miRNA and DNA, and could represent a potential bias. Finally, this retrospective cohort study allowed us to perform a careful selection of samples according to specific histological types. Although the samples were morphologically and molecularly similar, this selection constituted a bias, and may have influenced the performance of miRNA expression analysis. A confirmation on a prospective series of indeterminate thyroid nodules is therefore warranted. In this context, an RT-PCR assay for testing few target miRNAs would represent a practical and cost-effective strategy to be applied for validation purposes. Nonetheless, as shown by method comparison regression analysis, RT-PCR and nCounter data present an imperfect correlation, characterized by proportional differences, possibly due to technical biases related to reverse transcription and amplification processes. This aspect should be taken into account for the future selection of an appropriate validation methodology, along with a careful cost analysis.
